# Bilateral blindness after uneventful brolucizumab injection for macular degeneration

**DOI:** 10.1186/s12886-022-02305-1

**Published:** 2022-02-16

**Authors:** Ilan Barchichat, Michael Thiel, Oliver Job, Martin Schmid

**Affiliations:** Department of Ophthalmology, Cantonal Hospital of Lucerne, Spitalstrasse, 6000 Luzern-16, Switzerland

**Keywords:** Brolucizumab, Panuveitis, Perineural inflammation, Vasculitis, Bilateral blindness

## Abstract

**Background:**

We report a very severe case of bilateral panuveitis and ischemic vasculitis with possible perineural inflammation, which followed bilateral intravitreal brolucizumab administration in a patient with neovascular age-related macular degeneration (nAMD).

**Case presentation:**

On December 11, 2020, a 81-year-old woman presented with severe bilateral loss of vision. Eight days earlier, she had received uneventful bilateral injection of brolucizumab, a novel anti-vascular endothelial growth factor (VEGF) single-chain variable region (scFv) recombinant protein drug, for treatment of neovascular age-related macular degeneration (nAMD).

Slit-lamp examination revealed signs of a bilateral panocular vasculitis with ischemia. Scanning laser ophthalmoscopy of her left eye revealed marked vascular sheathing.

T1 fat-saturated post-contrast images of the orbit revealed a higher-than-normal signal of the choroid, with localized choroidal detachment. Additionally, pathologic enhancement was visible around the optic nerve in the orbit, which was interpreted as vasculitis. Due to the severe bilateral panuveitis with vasculitis, an additional vitreous tap was obtained, which revealed elevated levels of interleukin six and interleukin ten.

**Conclusions:**

To our knowledge, this is the first documented case showing both panuveitis and ischemic vasculitis with possible perineural inflammation. We do not recommend performing bilateral brolucizumab injections until more data is available regarding the mechanism of brolucizumab-induced vasculitis. From a clinical point of view, we find it difficult to justify the use of brolucizumab when there are other well-known agents, such as ranibizumab and aflibercept, which have better safety profiles and comparable efficacy.

## Background

Anti-VEGF drugs, such as ranibizumab and aflibercept, have been a widely used treatment for neovascular age-related macular degeneration (nAMD) and have generally been a safe procedure. Brolucizumab is the newest commercially available anti-VEGF agent for intravitreal injection, approved by the FDA in October 2019. It is a humanized single-chain antibody variable region fragment (scFV) that inhibits the binding of VEGF-A to VEGF receptors. However, within a few months post-marketing, several reports have described several adverse events, including severe retinal vasculitis.

Here we report a very severe case of bilateral panuveitis and ischemic vasculitis with possible perineural inflammation, which followed bilateral intravitreal brolucizumab administration in a patient with neovascular age-related macular degeneration (nAMD). Topical and systemic steroid treatment did not prevent progression to complete blindness in both eyes. To our knowledge, this is the first published case to demonstrate panocular and optic nerve ischemic involvement after brolucizumab injection. Practitioners should be aware of the potential for catastrophic adverse events with brolucizumab use, especially when used bilaterally.

## Case presentation

On December 11, 2020, a 81-year-old woman presented to the eye hospital of Lucerne, Switzerland, with severe bilateral loss of vision. Eight days earlier, she had received uneventful bilateral injection of brolucizumab, a novel anti-vascular endothelial growth factor (VEGF) single-chain variable region (scFv) recombinant protein drug, for treatment of neovascular age-related macular degeneration (nAMD). The patient had a 2-year history of bilateral nAMD, which had been treated with ten anti-VEGF injections (3 × bevacizumab, 7 × aflibercept) in her right eye, and 12 aflibercept injections in her left eye, with a final treatment interval of every 8 weeks. Her visual acuity was 20/200 in her right eye and 20/400 in her left eye. She had undergone a vitrectomy with membrane peeling for macular pucker, combined with cataract surgery, in the left eye in March 2016. Her relevant medical history included arterial hypertension, osteoporosis, and follicular thyroid carcinoma treated with radiotherapy years ago without metastasis. Current medications included aspirin cardio, euthyrox, lercanidipine, nebivolol, irbesartan, and ibandronate.

In September 2020, her treating ophthalmologist switched her anti-VEGF treatment to brolucizumab with the aim of reducing her clinical visits to once every 12 weeks, based on the published data of the HAWK and HARRIER trials [1]. Two weeks after her first same-day bilateral brolucizumab injections, the patient complained of a dark spot in her right peripheral visual field, which remained unchanged until her appointment 8 weeks later, at which she received her second same-day bilateral intravitreal brolucizumab injections. No additional examinations were performed to exclude signs of ocular vasculitis prior to the injections. A follow-up visit the next day revealed a mild anterior chamber reaction on the right eye, and no signs of inflammation on the left. No treatment was deemed necessary. Seven days later, the patient complained of moderate pain, redness, and blurry vision in both eyes. The following day, she presented to our emergency service due to severe vision loss in both eyes.

**Fig. 1:**
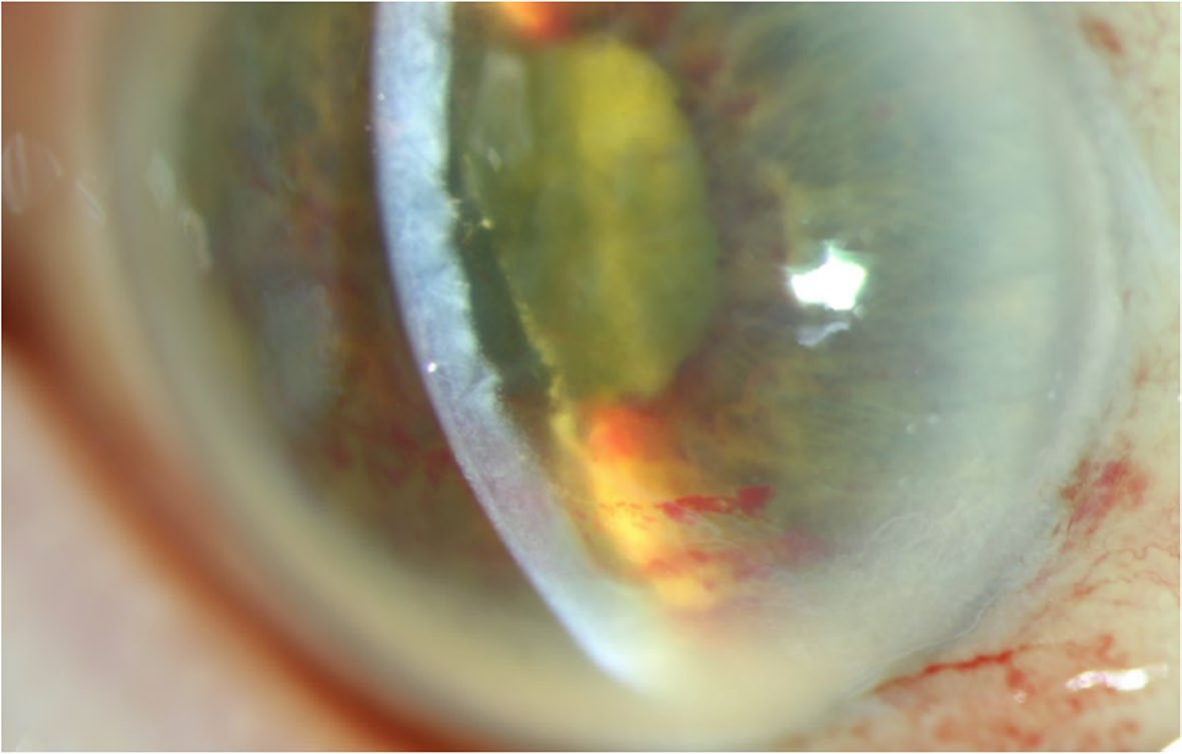
Slit-Lamp Photograph of Right Eye at 12 Days After the Second Brolucizumab Injection. The cornea presented with Descemet folds and severe stromal edema. The anterior chamber was filled with fibrin and dispersed blood on the corneal endothelium, without a hypopyon, and the iris presented with scattered small hemorrhages

**Fig. 2:**
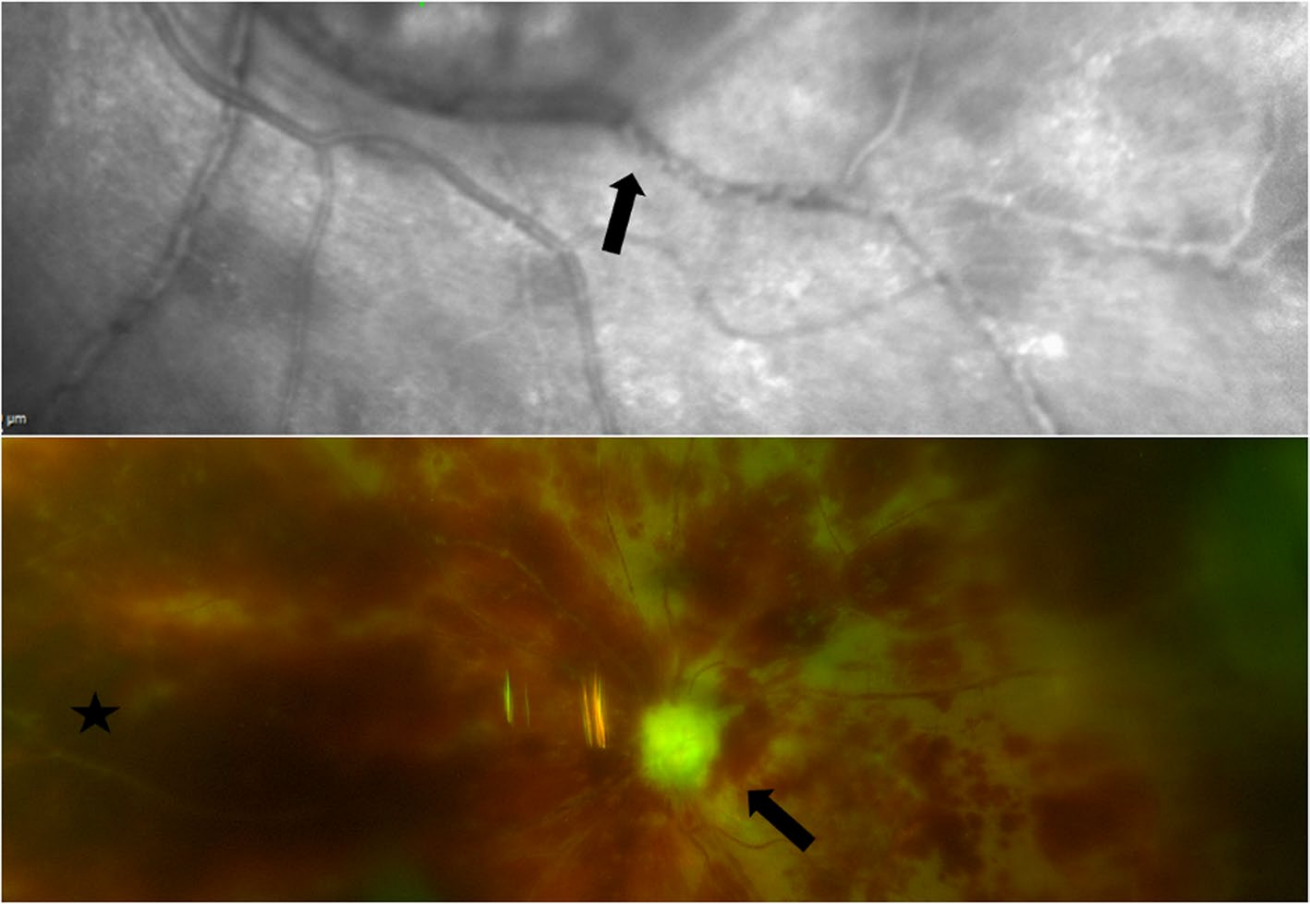
Laser Scanning Ophthalmoscopy and Fundusphotography. Top image shows laser scanning ophthalmoscopy of the left eye at 8 days after second brolucizumab injection. Arrow indicates signs of occlusive vasculitis. Bottom image shows fundusphotography at 40 days after second brolucizumab injection. Difficus retinal hemorrhages are visible. Black arrow indicates a completely atrophic optic nerve. Black star indicates vessel occlusions

At presentation, her visual acuity in each eye was classified as “hand motions”. Slit-lamp examination revealed marked conjunctivitis with diffuse hemorrhages in both eyes. The cornea of each eye exhibited Descemet folds and severe stromal edema. In both eyes, the anterior chamber was filled with fibrin and dispersed blood on the corneal endothelium, but without a hypopyon (Fig. 1), and the iris presented with scattered small hemorrhages. The intraocular pressure was 15 mmHg in her right eye and 8 mmHg in her left eye. The fundus of the right eye was not visible due to cataract and corneal edema, and her left eye exhibited diffuse opacities in the vitreous cavity and dense retinal hemorrhages. Scanning laser ophthalmoscopy of her left eye revealed marked vascular sheathing (Fig. 2)**.**

Based on the lack of hypopyon and an A- and B-scan sonography without hyperreflectivity in the vitreous cavity, infectious endophthalmitis was considered unlikely, and was later excluded since taps from the anterior chamber and vitreous cavity remained negative for eubacterial PCR and microbial culture assay. As the clinical picture resembled an ocular ischemic inflammatory syndrome, temporal arteritis and other forms of vasculitis were considered, but were deemed unlikely due to the patient’s history, lack of painful temporal arteries, and atypical laboratory results.

With a working diagnosis of brolucizumab-related vasculitis, immediate treatment was initiated with intravenous methylprednisolone (250 mg) per clinicians discretion, followed by oral prednisolone (1 mg/kilogram body weight). We decided against a complete pulse therapy cycle with IV methylprednisolone after a reviewing the literature that did not show any proven clinical benefit [2]. On the second day of treatment (10 days after second brolucizumab injection), her visual acuity had deteriorated to no light perception in both eyes and remained unchanged thereafter.

**Fig. 3:**
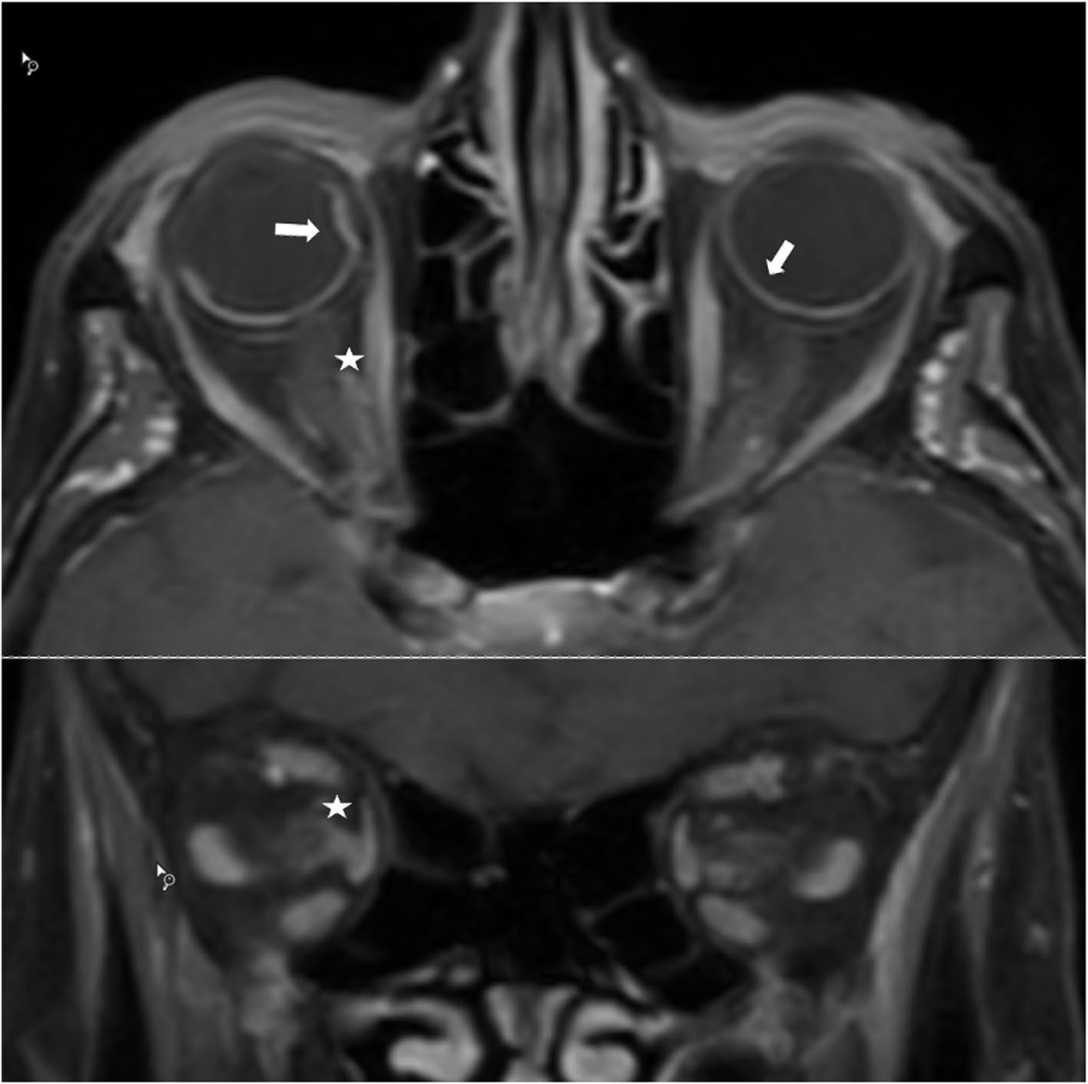
MRI T1 Fat-Saturated Post-Contrast Images of the Orbit. Arrow indicates higher than normal signal of the choroid with localized choroidal detachment. Asterisk indicates pathologic enhancement around the optic nerve in the orbit

MRI evaluations were performed to search for vasculitis outside the eyeball. T1 fat-saturated post-contrast images of the orbit revealed a higher-than-normal signal of the choroid, with localized choroidal detachment (Fig. 3). Additionally, pathologic enhancement was visible around the optic nerve in the orbit, which was interpreted as vasculitis [3]. Black blood MR sequences were also performed, which did not reveal CNS vessel involvement. Due to the suspected severe periocular vaculitis, an additional vitreous tap was obtained, which revealed elevated levels of interleukin 6 (> 300 pg/ml; normal vitreous value in control group: < 30 pg/ml) and interleukin 10 (40.6 pg/ml; normal vitreous value: < 3 pg/ml) [4]. Conjunctival biopsy of the left eye showed edema and inflammation, with intracapillary granulocyte accumulation. In the following days, intraocular pressure dropped to 1 mmHg in her right eye and 6 mmHg in her left eye, with signs of a bilateral panocular ischemia.

## Discussion and conclusion

Age-related macular degeneration (AMD) is a chronic progressive disease and a leading cause of vision loss [5]. Brolucizumab is the newest commercially available anti-VEGF agent for intravitreal injection (IVI). It inhibits the binding of VEGF-A to the receptors VEGFR1 and VGFR2 [6]. Brolucizumab (6 mg; Beovu, Novartis International AG, Switzerland) was approved for treatment of neovascular age-related macular degeneration in October 2019 by the US Food and Drug Administration (FDA), and in January 2020 by the Swiss authority for the licensing and monitoring of therapeutic products (Swissmedic). Phase III trials (HAWK and HARRIER) demonstrated that brolucizumab was noninferior to aflibercept in terms of preserving visual function at 48 weeks, and approximately 50% of eyes were maintained on a 12-week brolucizumab dosing interval with noninferior visual acuity outcomes compared to an 8-week aflibercept dosing interval [1]. Moreover, anatomic outcomes favored brolucizumab over aflibercept.

The HAWK and HARRIER studies concluded that brolucizumab was generally well-tolerated [1], with an ocular safety profile that was largely similar to the other approved anti-VEGF agents. However, within a few months post-marketing, reports have described several adverse events including severe retinal vasculitis and anterior segment inflammation, with up to 68 cases of unilateral blindness now reported to the WHO pharmacovigilance registry [7]. A subsequent re-evaluation of the approval studies revealed that among eyes treated with brolucizumab 6 mg, > 4% experienced intraocular inflammation (IOI) as adverse events, and 0.5% experienced IOI with retinal artery occlusion and visual loss of ≥ 15 EDTRS letters. This type of treatment complication has not been reported with the other currently approved anti-VEGF agents [8, 9]. Consequently, in June 2020, the FDA approved an updated brolucizumab label that includes additional safety information regarding retinal vasculitis.

Anti-VEGF drugs show notable variations in systemic pharmacokinetics and pharmacodynamics after intravitreal administration. Brolucizumab is a humanized scFv fragment with a molecular weight of 28 kDa. The other anti-VEGF drugs are larger molecules, with ranibizumab being a 50-kDa Fab fragment, and aflibercept a 115-kDa fusion protein. Ranibizumab, bevacizumab, and aflibercept are all rapidly cleared from the vitreous into the bloodstream. Brolucizumab shows significantly better tissue penetration compared to the other molecules [10, 11]. The Fab-fragment ranibizumab exhibits rapid renal clearance and short systemic exposure. Compared to conventional anti-VEGF drugs, the small molecular weight and the 6-mg dose of brolucizumab results in a 11- to 22-fold higher concentration of anti-VEGF antigen binding sites per injection. Together with the significantly better tissue penetration, this causes intensive VEGF blockade in the eye, and probably also in the periocular tissues [12].

All three anti-VEGF drugs are proteins and, despite their humanized structure, may cause immunogenicity [13]. In the last decade, immunogenicity has not been found to be a clinically relevant problem with the conventional anti-VEGF drugs. However, given the timely manner of the onset of severe inflammation in our patient, and the exclusion of other possible causes of severe intraocular inflammation, we assume that the blinding adverse effect of brolucizumab is likely due to occlusive vasculitis caused by either a delayed type IV hypersensitivity reaction to brolucizumab (similar to the first case of vasculitis reported by Haug et al. [14]), type III hypersentivity reaction [15–17] or by a toxic effect of massive VEGF depletion [18]. To further investigate this issue we performed a lymphocyte transformation test (LTT) which remained negative what excludes the possibility of a type IV hypersensitivity reaction. Hence, we speculate that a massive VEGF depletion or a type III hypersentivity reaction may be the most likely cause of the occlusive vasculitis observed in our patient.

Anti-VEGF drugs reportedly promote vasoconstriction, decreased blood flow velocity, and increased platelet aggregation [19, 20]. Due to its more potent anti-VEGF effect and its ability to easily penetrate tissue, brolucizumab may deplete VEGF to a degree that can cause damaging retinal arteriolar constriction, and subsequent occlusive vasculopathy of the choroid and adjacent tissue—a devastating effect that is not clinically observed with the weaker conventional anti-VEGF agents [18]. It is possible that the dark spot in our patient’s peripheral field of vision, which appeared after the first brolucizumab injections, might have also been caused by local occlusive vasculitis; however, this was not investigated by the injecting ophthalmologist before the second round of injections. Remarkably, the MRI scans of this case demonstrate that brolucizumab can potentially cause vasculitis not only in the retina, but also in the adjacent tissues, such as the choroid, iris, conjunctiva, and the optic nerve, resulting in a severe ocular ischemia. To our knowledge, this is the first documented case showing both panuveitis with ischemic vasculitis and possible perineural inflammation. The possibility of ocular ischemic syndrome can not be ruled out.

There is no general consensus regarding the treatment of severe occlusive vasculitis after brolucizumab injection. However, several publications discuss the administration of topical or systemic steroids. In mild cases, topical steroid eyedrops may be sufficient, while potent systemic corticosteroid treatment is recommended in severe cases [2]. In our case, the immediate initiation of intravenous therapy with methylprednisolone 250 mg, along with subsequent oral and topical corticosteroids, was clearly ineffective. In retrospect, in cases of severe vasculitis after brolucizumab treatment, it may be recommended to immediately start therapy with systemic anti-TNF-α and intravitreally administered corticosteroids, or even vitrectomy to reduce the cytokine load. However, based on the extraocular involvement and full-blown vasculitis in our case, it remains doubtful whether any treatment at that stage would have changed the outcome.

Bilateral treatment with anti-VEGF agents is common and is reportedly administered in over 30% of all AMD patients [21]. With conventional anti-VEGF drugs, bilateral treatment has appeared to be a safe procedure [22]. However, with brolucizumab, occlusive vasculitis occurs in ~ 0.5% of injections [1]. Therefore, bilateral treatment with brolucizumab may pose a high risk of blinding complications. To date, neither the US FDA nor the Swiss label advise against the bilateral application of brolucizumab. However, in November 2020, the Swiss Vitreoretinal Group (SVRG) recommended that brolucizumab should not be used in monocular patients. We speculate that our patient’s early complaints of a dark spot in her peripheral field of vision may have been a warning sign of vasculitis that was overlooked before the second injections, which were followed by severe bilateral panuveitis with vasculitis and possible perineural inflammation.

We do not recommend performing bilateral brolucizumab injections until more data is available regarding the mechanism of brolucizumab-induced vasculitis.

From our clinical point of view, we find it difficult in cases with bilateral intravitreal injections to justify the use of brolucizumab when there are other well-known agents, such as ranibizumab and aflibercept, which have better safety profiles and comparable efficacy.

## Data Availability

All data generated or analysed during this study are included in this published article.

## Abbreviations

AMD: Age-related macular degeneration
anti-VEGF: Anti-vascular endothelial growth factor
FDA: Food and Drug administration
Fig.: Figure
IOI: Intraocular inflammation
kDA: Kilodalton
LTT: Lymphocyte transformation test
MRI: Magnetic resonance imaging
nAMD: Neovascular age-related macular degeneration
scFV: Single-chain variable region
SVRG: Swiss Vitreoretinal Group
TNF-α: Tumor necrosis factor-alpha
VEGFR1/2: Vascular endothelial growth factor receptor 1/2
